# Transparent and Flexible Hierarchical Porous Structure of Polyvinyl Alcohol Aerogel: A Microstructure Study

**DOI:** 10.3390/ma17215312

**Published:** 2024-10-31

**Authors:** Xiaoli Li, Xuguang Zhang, Hexiang Zhang, Xiao Sun, Ying Mu, Thomas Barrett, Conor Doyle, Marilyn L. Minus, Yi Zheng

**Affiliations:** 1 Department of Mechanical and Industrial Engineering, Northeastern University, Boston, MA 02115, USA; li.xiaol@northeastern.edu (X.L.); zhang.xug@northeastern.edu (X.Z.); zhang.hex@northeastern.edu (H.Z.); sun.xiao1@northeastern.edu (X.S.); mu.yin@northeastern.edu (Y.M.); barrett.t@northeastern.edu (T.B.); doyle.con@northeastern.edu (C.D.); m.minus@northeastern.edu (M.L.M.); 2 Department of Chemical Engineering, Northeastern University, Boston, MA 02115, USA

**Keywords:** aerogel, transmittance, porous, optical

## Abstract

Aerogels have gained increasing attention due to their unique properties since their introduction in 1932. Silica aerogel, one of the earliest and most advanced types, is known for its high transparency and excellent thermal insulation. However, its internal pearl-like structure makes it extremely brittle, which limits its practical applications. To address this, through multiple refinements in formulation and production techniques, we developed a novel Polyvinyl Alcohol (PVA) aerogel using an innovative one-step standing method. This method significantly reduces the gelling time compared to the freeze–thaw method and eliminates the need for refrigeration, making it a more environmentally friendly and sustainable process. The resulting one-step standing PVA aerogel features a hierarchical porous structure, remarkable transparency, improved strength, and enhanced thermal insulation. Mechanical tests demonstrated that the PVA aerogel produced by the one-step standing method exhibited a significantly higher Young’s modulus of 4.2596 MPa, surpassing that of silica, copper nanowire (Cu NM), and graphene aerogels. Additional tests, including transmittance and thermal analysis, further confirmed that the one-step standing PVA aerogel excels in both transparency and thermal insulation. This combination of improved mechanical performance and light transmission opens novel potential applications, such as drug delivery systems, where the aerogel’s pore structure can store drugs while maintaining strength and transparency.

## 1. Introduction

When Kistler first introduced aerogels, a solid material with a highly porous and open structure, many different materials were used or considered as raw materials for aerogels [1]. This unique porous structure gives aerogels many distinct properties, such as thermal insulation, soundproofing, and adsorption. Choosing different substrates or manufacturing methods can alter these properties, which has led to the growing popularity of aerogels. The first aerogels demonstrated were silicon-based aerogels, most of which had a porosity of over 90%, making aerogels extremely low in density and commonly referred to as “solid air” [2]. At the same time as aerogels were first introduced, Kistler also introduced a new concept: supercritical drying. At the extension of the gas and liquid phases, a mixed phase exists, known as the supercritical phase, characterized by the critical temperature (T_C_) and pressure (P_C_). [3] When the system’s temperature and pressure exceed T_C_ and P_C_, respectively, it enters the supercritical phase [4]. The supercritical phase can be simply understood as a mixture of the gas and liquid phases, where there is no longer a distinct boundary between the two but rather a seamless integration [3]. Aerogels are derived from the drying of wet gels. The traditional method of obtaining wet gels is known as the sol–gel process [5]. It begins with the preparation of a solution, where solutes undergo physical or chemical cross-linking to form a three-dimensional network structure. After that, displacement and drying are carried out, leaving only the skeleton of the three-dimensional network structure, resulting in the porous aerogel [6,7,8,9]. Drying methods are simply divided into three types based on the drying environment: atmospheric drying; freeze drying; and supercritical drying. Each method has advantages and disadvantages, and researchers often choose the appropriate drying method based on the desired properties of the aerogel, budget, and other factors [10].

Silicon-based aerogels, one of the oldest and currently most developed types of aerogels, are renowned for their high transparency and extremely low thermal conductivity. Silica aerogel is a typical example. It is transparent with a light blue color and is very lightweight. Currently, it is being explored for use as thermal insulation [11], soundproofing [12], absorption [13], and optical materials [14]. However, it has not been widely commercialized due to its poor mechanical properties. Observations of the microstructure of silica aerogel reveal that its skeleton consists of silica particles linked together, resembling a series of pearl necklaces [15]. The connections between these “pearls” are relatively weak, and combined with the aerogel’s porous structure, this makes silica aerogel very brittle. Under certain pressures, it can break into small pieces or even powder. Researchers then began focusing on improving the mechanical properties of aerogels. At this point, polymers caught their attention. Polymers are large molecules with long-chain structures and come in a wide variety, possessing many unique properties, such as excellent mechanical strength, high transparency, flame retardancy, or thermal insulation. As a result, some researchers started using polymers to enhance the mechanical strength of silica aerogels [16,17]. Alternatively, polymers can be directly used as a matrix to produce carbon-based aerogels, such as polyimide [18,19], polyvinyl alcohol [20], and others [21]. From the properties of silica aerogels, it seems that excellent mechanical performance and transparency are mutually exclusive and difficult to achieve simultaneously. Therefore, this work aims to enhance mechanical strength while maintaining excellent transparency and preserving certain thermal insulation properties. Thermal insulation performance is contributed by the porous structure of the aerogel, while the excellent mechanical properties make the polymer stand out. Polyvinyl alcohol (PVA) exhibits good mechanical properties [22], water solubility [23], biocompatibility [24], and excellent optical performance [25]. The self-cross-linking ability and outstanding mechanical properties of PVA also allow for it to be made into fibers or films [26,27]. The high transparency of PVA film makes it a highly feasible material for this work.

In this work, PVA was selected as the raw material for the aerogel. No additional chemical cross-linking agents were added, and pure physical cross-linking was used. Supercritical drying was chosen as the drying method to maximize the retention of the three-dimensional porous structure. After several refinements, the final method is time-efficient and straightforward, producing transparent, flexible PVA aerogel sheets with consistent quality and excellent mechanical properties.

## 2. Materials and Methods

### 2.1. Materials

In this work, Dimethyl sulfoxide (CAS # 67-68-5, lot # 472301), purchased from Sigma-Aldrich Corporation (Burlington, MA, USA), was used as the solvent. The selected PVA is Mowiol 56–98 from Kuraray America Inc. (Chiyoda City, Japan), with a molecular weight of 105,600 g/mol, a degree of hydrolysis of 98.4 ± 0.4 mol%, and a residual acetyl content of 1.5 ± 0.4%. Methanol (CAS # 67-56-1, lot # A43420) was purchased from the Thermo Fisher Scientific (Waltham, MA, USA).

### 2.2. Methods

**Figure 1:**
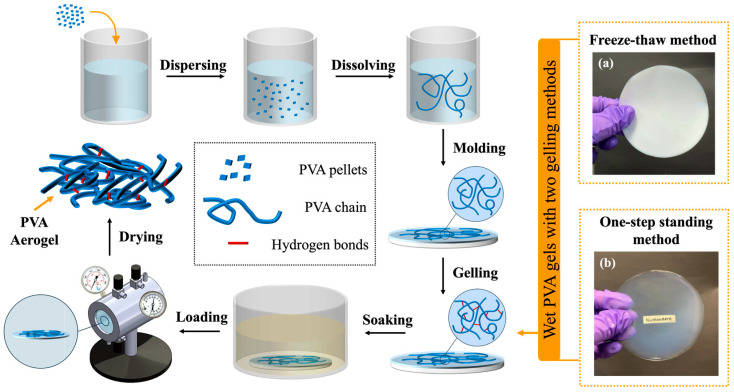
A schematic diagram of the PVA aerogel fabrication process: (**a**) wet gel prepared by freeze–thaw method; (**b**) wet gel prepared by the one-step standing method.

The PVA wet gel in this work is prepared using the classic sol–gel method. The liquid inside the wet gel is then removed through supercritical drying, leaving only the PVA aerogel composed of a three-dimensional PVA skeleton. Figure 1 shows a schematic diagram of the entire experimental process.

#### 2.2.1. Gelling Methods

The sol–gel method is a classic technique for preparing wet gels/hydrogels with the aim of transforming a solution into a gel. In this work, the side chains of PVA are hydroxyl groups, which can form hydrogen bonds when they come close to each other, resulting in physical cross-linking. Therefore, no additional chemical cross-linking agents are introduced in this process. Initially, PVA pellets are slowly added to the solvent DMSO, then heated and stirred to fully dissolve the PVA into the DMSO, forming a completely transparent PVA solution. The solution is then transferred into a mold for the subsequent sol–gel transition. The choice of mold can vary depending on the intended application. In this work, the mold selection underwent several iterations. Initially, a commercially purchased glass dish was used, but because the bottom of the dish was not flat enough, it led to uneven thickness in the final product. A chemically safe PTFE dish was then made, but due to the high viscosity of the polymer solution, the solution “crawled” up the walls of the dish, resulting in thicker edges and a thinner center in the final product. Finally, a custom-made PTFE plate was selected as the mold for forming thin sheet PVA aerogels, which allowed for the stable formation of thin aerogels with uniform thickness. The core idea of the sol–gel transition process is to use a gelling method to promote cross-linking within the solution. A commonly used method is the freeze–thaw method. As the name suggests, this method helps the solution cross-link through cycles of freezing and thawing.

After transferring the solution into the mold, both the mold and the solution can be placed in a freezer for freezing. The freezing temperature needs to be set below the solvent’s melting point to ensure that the movement of the solution is restricted. The purpose of freezing is to limit the movement of the polymer chains by freezing the solvent, giving the -OH groups that are already close to each other enough time to cross-link into hydrogen bonds. The sample is then thawed at room temperature to restore chain movement, providing additional opportunities for other -OH groups that have not yet cross-linked to come close and form hydrogen bonds. After several freeze–thaw cycles, the PVA chains in the solution have cross-linked into a three-dimensional network structure. In this work, the freeze–thaw process involves freezing at 4 °C for 24 h, followed by thawing at room temperature for 12 h, repeating the cycle twice, and finally freezing again for 24 h before immersing the sample, along with the mold, in methanol, a poor solvent for PVA. Thus, the freeze–thaw process takes 4 days to complete, and the resulting gel is opaque, as shown in Figure 1a. This work introduces an innovative one-step standing method, which allows the PVA to cross-link while maintaining higher transparency, saving both time and energy, making it a more eco-friendly and sustainable approach. The specific procedure involves placing the mold and sample at room temperature for self-cross-linking for 24 h, followed by direct immersion in methanol. This significantly reduces the time required and results in transparent samples, as shown in Figure 1b. The purpose of soaking in methanol during this step is to replace the good solvent DMSO with a poor solvent for PVA, preventing the PVA from dissolving in DMSO. This allows the three-dimensional PVA network structure to be preserved, enabling the wet gel to proceed to the drying.

#### 2.2.2. Drying Methods

The drying process is essential for aerogels. The name “aerogel” comes from the fact that the drying process allows gas to replace the liquid in the wet gel. Common drying methods include ambient pressure drying, freeze-drying, and supercritical carbon dioxide drying. Each of these methods has advantages and disadvantages, and researchers typically choose based on the project’s requirements. According to the classic phase diagram, these three methods can be distinguished. Ambient pressure drying directly transforms the substance from the liquid phase to the gas phase, crossing the liquid–gas boundary. However, the downside of crossing this boundary is the presence of surface tension, which can cause some collapse in the three-dimensional network structure. To avoid crossing the pressure boundary directly, freeze-drying and supercritical drying have emerged as alternatives. As the name suggests, freeze-drying involves freezing the substance from the liquid phase to the solid phase and then transitioning to the gas phase. This avoids crossing the liquid–gas boundary, but it still requires crossing the solid–gas boundary. During this transition, volume changes can occur, leading to some degree of structural collapse. Additionally, due to the freezing process, as in the freeze–thaw cycle of the sol–gel process, freeze-drying can result in a loss of transparency in PVA aerogels. Supercritical carbon dioxide drying, on the other hand, raises the temperature and pressure beyond the substance’s critical temperature and pressure, reaching a supercritical phase, which is a mixture of liquid and gas. The transition to the gas phase from this supercritical phase avoids crossing any phase boundaries, thus preventing unwanted structural collapse. For this reason, supercritical carbon dioxide drying was chosen in this work, as it also effectively preserves the transparency of the aerogel.

### 2.3. Characterizations

#### 2.3.1. Scanning Electron Microscope (SEM)

A Zeiss Supra 25 scanning electron microscope, with an accelerating voltage of 5 kV, was used to image the internal structure of the aerogel. A Gatan high-resolution ion beam coater was used to coat all sample surfaces with a thin layer of conductive metal. After freezing with liquid nitrogen, the samples were fractured to obtain cross-sections, ensuring that the cross-sections remained intact without structural collapse caused by external forces.

#### 2.3.2. The Analysis for Recording Weight Loss During Heating

To assess the aerogels’ thermal properties, thermogravimetric analysis was performed using a Q50 from TA Instruments (New Castle, DE, USA). This technique measures changes in sample mass as a function of temperature. In this study, this analysis was utilized to compare the thermal characteristics of PVA pellets, films, and aerogels.

#### 2.3.3. Dynamic Mechanical Analyzer (DMA)

Tensile tests were performed on PVA aerogels using a dynamic mechanical analyzer (2710-103 series, INSTRON Instrument, Norwood, MA, USA). The obtained Young’s modulus was used to compare with that of other existing aerogels.

#### 2.3.4. Spectrophotometer (V-770)

The transparency of the aerogels was assessed using a JASCO V-770 UV-visible/NIR spectrophotometer (Easton, MD, USA), which covers a wavelength range of 300 nm to 2500 nm. The instrument operated with a scanning speed of 200 nm/min and a UV/VIS bandwidth of 5 nm.

## 3. Results and Discussion

### 3.1. Mechanical Property

**Figure 2:**
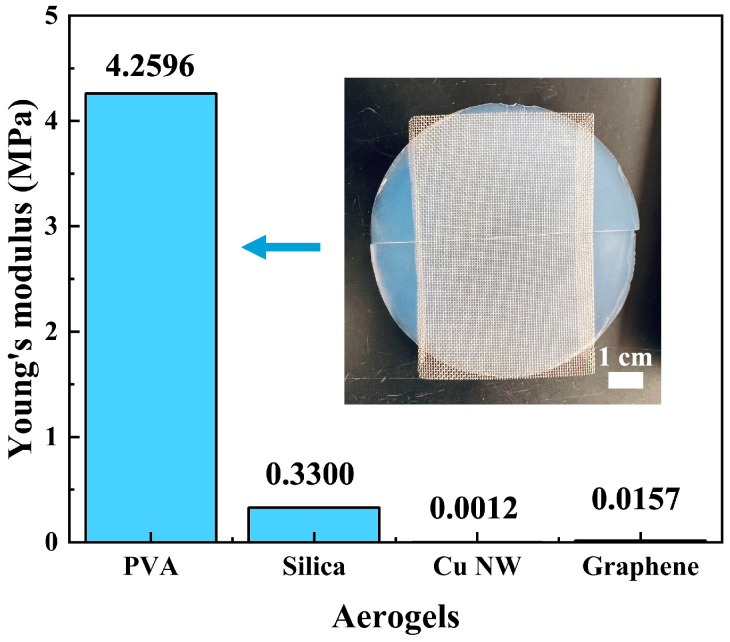
Young’s modulus of different aerogels, including PVA aerogel (this work), silica aerogel [28], Cu NW aerogel [29], and graphene aerogel [30]. Inset: transparent PVA aerogel produced through the one-step standing method.

Compared to silica aerogels, this work focuses on enhancing mechanical performance while maintaining transparency. Therefore, tensile tests were conducted on the samples produced using the one-step standing method, and the obtained Young’s modulus was compared with that of silica aerogels, Cu NW aerogels, and graphene aerogels from the literature [28,29,30]. The samples were prepared by cutting long, thin strips from the PVA aerogel sheets using a new blade from top to bottom. The strips were approximately 10 mm in length, with the thickness corresponding to the thickness of the aerogel sheet, and the width was around 1–2 mm. Tensile tests were performed at room temperature using the 2710-103 series INSTRON Instrument. Cutting from top to bottom in one motion ensured that defects caused by the cutting process were avoided, ensuring accurate data. More than 10 valid tests were conducted, and the average value was taken to improve data accuracy. As shown in Figure 2, the Young’s modulus of the aerogels produced in this work is significantly higher than the other three. Additionally, the inset shows that the PVA aerogel is transparent with a light blue tint. Videos S2 and S3 showcase the manual bending of a one-step standing PVA aerogel and a commercially purchased silica aerogel. The one-step standing PVA aerogel demonstrates excellent flexibility due to the properties of the polymer material, allowing it to return to its original shape after bending. In contrast, the brittle silica aerogel breaks apart when pressure is applied with fingertips. Additionally, Video S1 illustrates the manual bending of PVA aerogels over 200 times, providing a more intuitive representation of the flexibility of PVA transparent aerogels. 

### 3.2. Morphology

**Figure 3:**
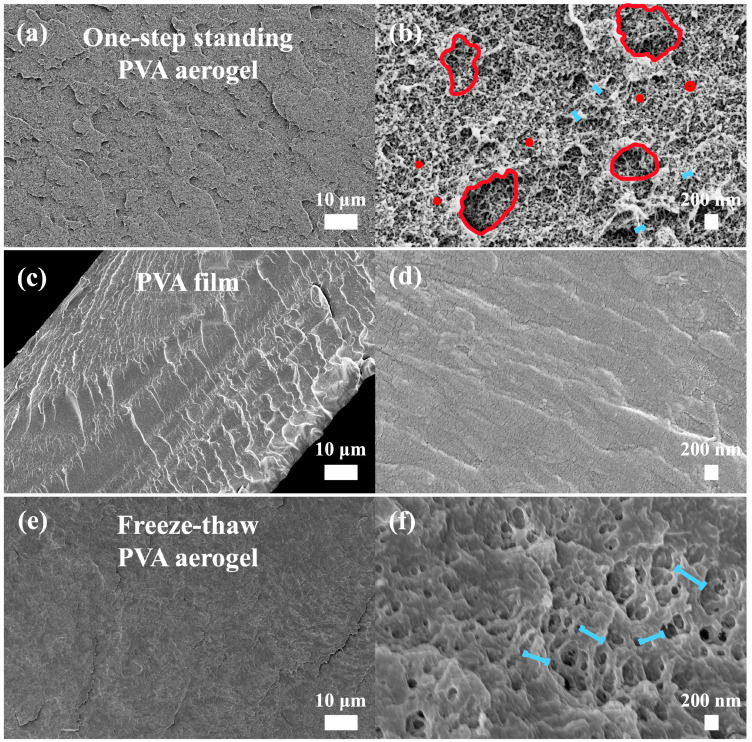
SEM images of the internal structure of PVA products. (**a**,**b**) SEM images of the internal structure of PVA aerogels produced using the one-step standing method, corresponding to magnifications of 500× and 20,000×, respectively. (**c**,**d**) SEM images of the internal structure of PVA films produced using the one-step standing method, corresponding to magnifications of 500× and 20,000×, respectively. (**e**,**f**) SEM images of the internal structure of PVA aerogels produced using the freeze–thaw method, corresponding to magnifications of 500× and 20,000×, respectively. (The red circles indicate the pores, and the blue lines mark the diameter of the skeleton, which is considered the “particle size” in the text).

A scanning electron microscope was used to observe and image the PVA aerogels dried by supercritical carbon dioxide drying and the PVA film dried by ambient pressure drying. Different drying methods were applied to study how they affected the internal structure. To observe a clear and accurate representation of the internal structure, an intact cross-section is essential. However, common cutting methods, which apply downward pressure during slicing, were avoided as they could collapse the internal structure. Additionally, PVA products are flexible and difficult to fracture directly. In this work, liquid nitrogen at a temperature as low as −195 °C was used to freeze the samples, making them brittle and allowing for clear fractures to obtain undamaged cross-sections. Theoretically, supercritical drying preserves the three-dimensional porous structure of the wet gel well, as mentioned earlier, because no phase boundary is crossed, avoiding volume changes and surface tension. As shown in Figure 3a, under 500× magnification, the internal structure of the PVA aerogel appears more uniform and porous. In contrast, the PVA film at the same magnification, as depicted in Figure 3c, looks very dense. Increasing the magnification to 20,000×, Figure 3b reveals an intriguing hierarchical porous structure within the aerogel. As highlighted by the red circles, numerous small pores exist within the larger pores. The small pores are measured in the tens of nanometers, while the larger pores are measured in the hundreds of nanometers. This hierarchical porous structure opens up various applications for PVA aerogels. For instance, the large pores could be used for drug delivery, providing more space for drug storage or adsorption, while the small pores help maintain the stability of the three-dimensional structure, making it less prone to collapse. In contrast, the internal structure of the PVA film shown in Figure 3d does not reveal any obvious porous structure. This reveals that the ambient pressure drying process nearly destroys all the porous structures of the PVA wet gels.

The internal structure of the polymer-based aerogels consists of a three-dimensional network made of polymer chains, so the particle size can be considered the width of the framework. Figure 3a,b shows the internal structure of transparent aerogels produced using the one-step standing method, while Figure 3e,f depicts the internal structure of opaque aerogels made using the freeze–thaw method. All four images reveal a porous structure, and a comparison of Figure 3b,f highlights a significant difference: the samples produced by the freeze–thaw method have a larger particle size, marked by the blue lines, on the order of hundreds of nanometers. In contrast, the particle size of the samples made using the one-step standing method is in the range of tens of nanometers.

From an optical perspective, scattering can be classified into two types: Mie scattering and Rayleigh scattering. Mie scattering occurs when the particle size is equal to or greater than the light wavelength, which explains why fog appears opaque and white. Rayleigh scattering, on the other hand, occurs when the particle size is much smaller than the light wavelength, which accounts for the blue color of the sky. Considering the particle sizes of the PVA aerogels, the samples produced by the freeze–thaw method are opaque, consistent with Mie scattering, while those made using the one-step standing method are transparent and appear light blue, aligning with Rayleigh scattering.

### 3.3. Transmittance

**Figure 4:**
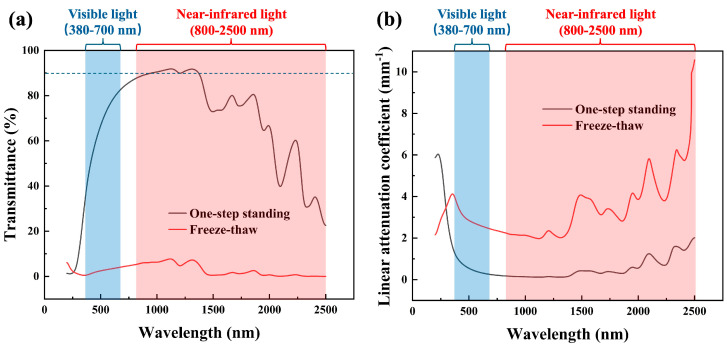
(**a**) The transmittance curves for PVA aerogels made using the one-step standing method and freeze–thaw method. (**b**) The linear attenuation coefficient curves for PVA aerogels made using the one-step standing method and freeze–thaw method.

This work used a V-770 UV-visible/NIR spectrophotometer to perform transmittance tests on aerogels produced by the one-step standing method and those produced by the freeze–thaw method. The wavelength range was set from 300 to 2500 nm, covering visible light and near-infrared light. The scanning speed was set at 200 nm/min. As shown in Figure 4a, the transmittance of the aerogels made using the one-step standing method is significantly higher than those made by the freeze–thaw method. However, transmittance is not only related to the material and internal structure but also to the thickness of the sample. Theoretically, any material with an infinitely small thickness would allow light to pass through. Therefore, in this work, Beer–Lambert Law, as represented by Equation (1), was applied to eliminate the effect of thickness on transmittance [31]. In the equation, T represents the transmittance of the material [%], α is the linear attenuation coefficient [mm^−1^], and x represents the sample thickness [mm]. The linear attenuation coefficient measures how challenging it is for physical entities like photons, sound waves, or electrons to travel through a material in one direction. For evaluating material transparency, a higher coefficient means that light diminishes quickly as it passes through, while a lower coefficient indicates easier light transmission. In other words, a smaller value reflects greater transparency.

(1) $$T=e^{-\alpha x},$$

As shown in Figure 4b, calculations reveal that the one-step standing PVA aerogel still possesses superior light transmission compared to PVA aerogel made using the freeze–thaw method. This perfectly aligns with the earlier discussion about how particle size determines the scattering mechanism in a material. The one-step standing PVA aerogel follows Rayleigh scattering, resulting in better transparency and exhibiting a light blue color.

### 3.4. Thermal Stability

**Figure 5:**
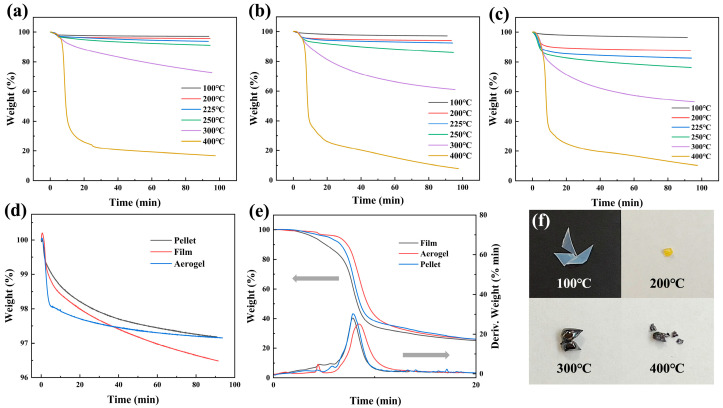
(**a**) the curve of weight percentage versus time for one-step standing PVA aerogel, (**b**) for pure PVA pellet, (**c**) for PVA film; (**d**) comparison curves of pure PVA pellet, PVA film, and one-step standing PVA aerogel at 100 °C; (**e**) comparison curves and derivative curves of pure PVA pellet, PVA film, and one-step standing PVA aerogel at 400 °C; (**f**) PVA aerogel residue after heating under 100 °C, 200 °C, 300 °C, and 400 °C. (The gray arrows in (**e**) indicate the corresponding axis meanings for the curves).

PVA pellets, PVA films (dried at ambient pressure), and one-step standing PVA aerogels (dried supercritically) were heated in a furnace under an air environment, maintaining the target temperature for a period to ensure that the weight loss occurred completely at that temperature. For each sample, thermal analysis was performed at different temperatures. As shown in Figure 5a–c, comparing the curve of weight percentage versus time of these three sample types at the same temperature reveals that aerogel has the highest remaining weight percentage. This means that the thermal performance of PVA aerogels is superior to both PVA pellets and PVA films. The comparison curve at 100 °C in Figure 5d shows that the aerogel’s weight percentage decreases sharply in the initial stage. This is because, at lower temperatures, the initial weight loss is primarily due to the evaporation of absorbed moisture, and the porous structure of the aerogel facilitates the removal of moisture. Therefore, the weight loss decreases significantly and stabilizes at the lower temperature range. Figure 5e presents the weight loss curves and corresponding derivative curves for the three types of PVA products. It can be observed that the derivative curve of the PVA aerogel shifts to the right compared to those of the pellet and film, indicating that the aerogel exhibits superior thermal stability among the three samples. Figure 5f displays the residues of the PVA aerogel after heating at different temperatures. Based on the color, it is clear that heating at 100 °C did not cause the aerogel to lose its porous structure, as it still appears light blue. As the temperature increases, the aerogel gradually carbonizes. Table 1 shows the remaining weight percentage of the three samples after being held at different temperatures in a furnace for 90 min. These correspond to the lowest points of each curve in Figure 5a–c. The table demonstrates that at 200 °C, 225 °C, 250 °C, 300 °C, and 400 °C, the remaining weight percentage of the PVA aerogel is the highest, indicating that the aerogel has better thermal stability.

**Figure 6:**
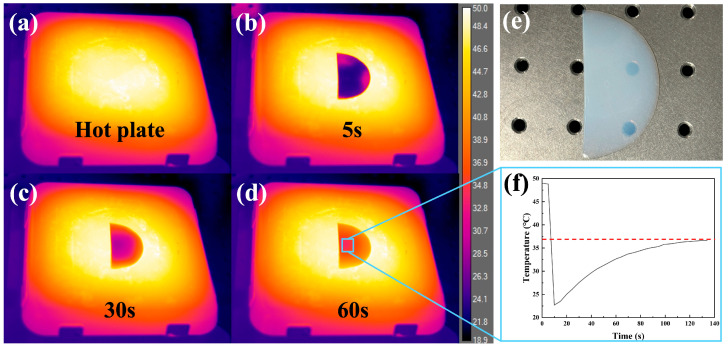
Thermal images of one-step standing PVA aerogel on a 50 °C hot plate: (**a**) hot plate without the sample; (**b**) hot plate with the sample after 5 s; (**c**) hot plate with the sample after 30 s; (**d**) hot plate with the sample after 60 s; (**e**) the sample after the heating test; and (**f**) the curve of the temperature versus time of the selected area shown in (**d**).

In exploring the thermal properties of PVA aerogels, direct heating tests were conducted, and a thermal camera was used to record the temperature of the aerogel. The direct heating test was conducted with 50 °C as the safe temperature. The sample was placed on a hot plate preheated to 50 °C, and the process was recorded using a thermal camera. The image of the preheated hot plate under the thermal camera was captured and shown in Figure 6a, where it can be seen that the hot plate has reached the target temperature of 50 °C. Subsequently, thermal images of the sample placed on the heated hot plate for 5 s, 30 s, and 60 s were shown in Figure 6b–d. It can be observed that, despite the sample being very thin, it still maintains a certain temperature difference with the hot plate, demonstrating the thermal insulation capability of the aerogel. Figure 6e shows the appearance of the sample after the test, which retained its transparency and light blue color, indicating that the internal porous structure was still intact. The curve shown in Figure 6f represents the temperature variation within a fixed area, which is marked by a blue box in Figure 6e. At the start of the curve, the temperature can be read as having already reached 50 °C. After placing the aerogel at room temperature, a sharp drop in temperature to room temperature is observed. Over time, the temperature eventually stabilizes at 36.6 °C, reaching a plateau while maintaining a temperature difference of over 10 °C, providing a more intuitive demonstration of the aerogel’s thermal insulation capability.

## 4. Conclusions

Aerogels have recently attracted significant attention due to their unique properties, and a wide range of aerogels with various substrates have been developed. Among them, silica aerogel, the earliest and most established, stands out for its exceptional transparency and thermal insulation. However, its pearl necklace-like internal structure makes it highly brittle, severely limiting its practical applications. This research addresses this challenge by developing a novel approach to enhance the mechanical properties of aerogels without compromising their transparency. In this work, polyvinyl alcohol (PVA), a versatile polymer known for its self-cross-linking capabilities and long molecular chains, was chosen as the raw material. Unlike traditional aerogels, this study introduces a groundbreaking one-step standing method, a significant innovation in aerogel fabrication. This new method drastically reduces the gelling time compared to the conventional freeze–thaw method and eliminates the need for external cooling equipment, making it both energy-efficient and eco-friendly. The result is a mechanically superior aerogel that retains high transparency, offering a promising new material for sustainable applications.

Several characterizations were performed afterward. Tensile testing using DMA revealed that the Young’s modulus of the one-step standing PVA aerogel is 4.2596 MPa, significantly higher than that of silica aerogels, Cu NW aerogels, and graphene aerogels reported in the literature. SEM observations of carbon dioxide supercritical-dried PVA aerogel and ambient pressure-dried PVA film showed that the aerogel had a porous internal structure. A comparison between the one-step standing PVA aerogel and the freeze–thaw PVA aerogel revealed that the freeze–thaw aerogel had larger particle sizes in the hundreds of nanometers, consistent with Mie scattering. In contrast, the one-step standing aerogel exhibited a hierarchical porous structure, where the small-pore structure is nested within the skeletal framework of the large pores. The small pores measured tens of nanometers, consistent with Rayleigh scattering, which explains why it is transparent and exhibits a light blue color. This unique hierarchical pore structure opens up more possibilities for applications, such as drug delivery, where the large pores can store more drugs, and the small pores can ensure excellent mechanical performance while maintaining transparency. Further transmittance testing on the aerogels produced by the two types of gelling methods provided a more direct comparison of their effects on transparency. Since transparency is not only related to the material but also closely related to sample thickness, the parameter representing light transmission capability was calculated using Beer–Lambert law. The results indicated that the one-step standing PVA aerogel continued to exhibit superior light transmission, which is consistent with the SEM observations. Such a flexible and transparent material has potential applications in areas like solar energy conversion. Solar energy conversion devices often require materials that can allow sunlight to penetrate while also providing some thermal insulation to reduce energy loss. Aerogels have been considered for this photothermal application, but their brittle structure has limited their development. Therefore, the final product of this work has a certain potential in the photothermal industry. Subsequent thermal performance tests were conducted using the method for recording weight loss during heating to examine PVA pellets, PVA aerogels, and PVA films. By comparing the weight loss curves at different temperature plateaus, the results showed that aerogel, indeed, had better thermal insulation properties. A comparison of the three curves at 100 °C revealed that the aerogel curve dropped more sharply at the beginning, further confirming the existence of its porous structure.

## Figures and Tables

**Table 1:** PVA aerogel, PVA pellet, and PVA film weight (%) after 90 min at various temperatures.

Samples	100 °C	200 °C	225 °C	250 °C	300 °C	400 °C
PVA aerogel	97.15%	95.68%	93.80%	91.11%	72.79%	16.77%
PVA pellet	97.17%	94.01%	92.39%	86.02%	61.05%	7.91%
PVA film	96.48%	87.75%	82.57%	76.29%	53.16%	10.38%

## Data Availability

The original contributions presented in the study are included in the article/Supplementary Materials, further inquiries can be directed to the corresponding author.

## Supplementary Materials

The following supporting information can be downloaded at https://www.mdpi.com/article/10.3390/ma17215312/s1, Video S1: pva aerogel bending; Video S2: pva aerogel; Video S3: silica aerogel.
